# Varicella Zoster Virus and Stroke: An Intricate Relationship

**DOI:** 10.3390/v17121591

**Published:** 2025-12-08

**Authors:** Achilleas Livieratos, Lars Erik Schiro, Charalambos Gogos, Georgios Ntaios, Karolina Akinosoglou

**Affiliations:** 1 Independent Researcher, 15238 Athens, Greece; 2 Independent Researcher, 0284 Oslo, Norway; larserik.schiro@outlook.com; 3 Department of Medicine, University of Patras, 26504 Rio, Greece; cgogos@med.upatras.gr (C.G.); akin@upatras.gr (K.A.); 4 1st Medical Propedeutic Department of Internal Medicine & Stroke Unit, AHEPA Hospital, Aristotle University of Thessaloniki, 54124 Thessaloniki, Greece; gntaios@auth.gr; 5 Department of Internal Medicine and Infectious Diseases, University General Hospital of Patras, 26504 Rio, Greece

**Keywords:** Varicella Zoster Virus, stroke, vasculopathy

## Abstract

Varicella Zoster Virus (VZV), responsible for chickenpox and herpes zoster, has emerged as a significant contributor to cerebrovascular disease. Mounting evidence indicates that VZV reactivation may precipitate ischemic and hemorrhagic stroke through mechanisms of viral vasculopathy, immune evasion, and vascular inflammation. While antiviral therapy remains the cornerstone of treatment, several adjunctive regimens exhibit encouraging results in controlling endothelial inflammatory response. This targeted review synthesized findings from 31 studies, including clinical cohorts, in vitro models, and pathological analyses, to evaluate the relationship between VZV and stroke, with emphasis on treatment management beyond antivirals. Evidence demonstrates that VZV antigens are frequently detected within cerebral arteries, where they induce transmural inflammation, endothelial dysfunction, and thrombosis, thereby increasing stroke risk, particularly in the weeks following herpes zoster. Adjunctive therapies such as corticosteroids, statins, and resveratrol show promise in mitigating vascular inflammation, though clinical validation is limited. Preventive measures, especially zoster vaccination, significantly reduce herpes zoster incidence and may lower subsequent stroke risk, yet global uptake remains insufficient. Collectively, the data underscore the need for improved diagnostic tools, combination treatment strategies, and expanded vaccination programs to address the substantial public health burden of VZV-associated stroke.

## 1. Introduction

Varicella Zoster Virus (VZV), a highly contagious human herpesvirus, is nearly ubiquitous worldwide, with over 90% of adults showing serologic evidence of prior infection, particularly in temperate regions [1]. VZV manifests primarily as chickenpox in children prior to adolescence, while herpes zoster (shingles), caused by viral reactivation, disproportionately impacts older and immunocompromised populations. Global estimates suggest that herpes zoster is now increasing due to aging populations and immune suppression despite access to antivirals, with lifetime risk of herpes zoster at approximately 30%, making it one of the most common viral reactivations worldwide [2,3]. Despite estimated direct medical costs reaching $2.4 billion annually in the United States, with comparable cost levels in Europe and Asia, only 18% of countries worldwide have implemented universal VZV vaccination programs, with shingles vaccine uptake remaining low in regions with high disease prevalence [3]. However, accumulating evidence highlights a potentially broader disease spectrum that may include serious vascular and neurological complications, such as ischemic and hemorrhagic strokes, even in the absence of rash [4,5].

VZV’s neurotropism and potential vasculotropic interactions implicate it in a growing number of stroke syndromes, especially among vulnerable populations [5,6]. Meta-analyses report the first couple of weeks post-zoster as the period of increased occurrence, which remains marginally elevated one year post-infection [7]. Ophthalmic zoster, in particular, carries a higher cerebrovascular risk, with a relative stroke risk often exceeding twice that of more distant infections due to anatomical proximity to cerebral vessels [8,9]. Pediatric populations are also at higher risk of focal cerebral arteriopathy, frequently involving the middle cerebral artery, following primary varicella infection [10,11]. Given the high global prevalence of latent VZV infection and the public health burden of stroke, elucidating this relationship is critical [6].

Both experimental and pathological studies provide mechanistic insights into VZV vasculopathy. Analyses on preclinical models showed that the transport of alphaherpesviruses, such as pseudorabies virus, from ganglia to vasculature, depends on axonal kinesin motors and specific viral proteins such as gE, gI, and US9 [12]. Findings from human studies support this trajectory, with post-mortem analyses and blood vessel biopsies frequently revealing the presence of VZV DNA, RNA, or protein antigens within affected arteries [13,14,15]. Vascular damage resulting from VZV infection has been found to involve transmural inflammation, intimal hyperplasia, endothelial dysfunction, thrombosis, and arterial remodeling [16,17,18]. These pathological features result in a wide range of vascular events such as large artery atherosclerotic stroke and small vessel disease strokes, aneurysms, and intracerebral hemorrhages [16,17,18]. Studies using black-blood MRI have further enhanced diagnostic accuracy by revealing inflammatory changes within blood vessel walls [9,19]. Despite the availability of novel and more advanced diagnostic tools, confirmation of a causative relationship between VZV and stroke remains elusive in many cases. The absence of classic symptoms, such as rashes, CSF pleocytosis, or detectable viral DNA, further complicates accurate diagnosis. Anti-VZV antibodies within the CSF are the most accurate and reliable marker of ongoing infection and should be prioritized in suspected cases of VZV vasculopathy [20]. Other promising biomarkers include thrombotic markers such as D-dimer and β-thromboglobulin, which may help guide adjunctive therapies but do not confirm VZV presence or interaction [14]. Systemic inflammation-related biomarkers are also relevant, as elevations in IL-6, TNF-α, and other proinflammatory cytokines after VZV reactivation align with the recognized prothrombotic state that accompanies herpes zoster [15].

While intravenous acyclovir, often accompanied by corticosteroids to mitigate inflammation, remains a central piece of the arsenal used to combat VZV infection and recurrence, a growing body of research has explored alternative and adjunctive treatments to manage VZV infection and its complications [21,22,23]. Both live-attenuated and recombinant subunit zoster vaccines significantly reduce the incidence and severity of herpes zoster and its long-term complication, postherpetic neuralgia [23,24]. For exposed immunocompromised patients, passive immunization with varicella zoster immune globulin has been shown to offer temporary protection and can prevent severe disease progression [24,25]. Anti-inflammatory therapies, such as corticosteroids, have been used as adjuncts to reduce acute neuritis and may help mitigate the vascular inflammation linked to VZV-associated stroke, although their use remains controversial due to immunosuppressive risks [18,26]. Compounds such as resveratrol have been proposed as potential neuroprotective agents against VZV-induced vasculopathy, though clinical validation of resveratrol is still pending [27,28,29]. Management of postherpetic neuralgia also includes neuromodulatory treatments such as gabapentin, pregabalin, and tricyclic antidepressants, which can be more effective than antivirals alone for long-term pain control [30,31]. Additionally, emerging genetic approaches such as CRISPR/Cas9 gene editing and locked nucleic acid antisense therapies are being investigated to directly inhibit VZV gene expression and viral replication, offering promising future avenues beyond pharmacologic suppression [32,33]. Together, these therapies represent an evolving landscape of multi-modal strategies for managing VZV infection beyond traditional antivirals [34,35].

The clinical, pathological, and mechanistic evidence for a causal relationship between VZV and stroke is growing; however, diagnostic challenges and treatment resistance persist. We performed a targeted literature review to examine the relationship between VZV and stroke, with emphasis on epidemiology, pathophysiological mechanisms, experimental and clinical models, and therapeutic strategies, especially among non-antivirals. We also considered non-antiviral management approaches, including agents with anti-inflammatory or vasoprotective effects.

## 2. Materials and Methods

Literature research was conducted using PubMed, Embase, and Scopus up to November 2025, using the keywords *“Varicella Zoster Virus” AND “stroke” AND “vasculopathy” OR “anti-virals”*.

The inclusion criteria focused on studies that (a) reported clinical, experimental, or imaging evidence linking VZV to stroke or cerebrovascular disease; (b) investigated therapeutic strategies, including antivirals, non-antiviral agents (e.g., corticosteroids, immunomodulators), or vaccines; (c) examined experimental models (in vitro, in vivo, or clinical) addressing VZV replication, vascular inflammation, or neurological outcomes; and (d) explored mechanistic pathways such as immune suppression, endothelial dysfunction, vascular remodeling, and inflammatory responses. Eligible studies included original research on human subjects, animal models, and cell-based assays, provided they were published in English and in peer-reviewed journals. Studies were excluded if they focused solely on dermatologic manifestations, lacked relevance to vascular or neurological outcomes, involved non-VZV viruses without comparison to VZV, or were unavailable in full text. Only English studies were included, while duplicates were eliminated. Three independent reviewers screened articles and resolved disagreements through discussion and consensus. The final analysis aimed to synthesize multidisciplinary evidence to inform future research and clinical management of VZV-related stroke.

**Figure 1:**
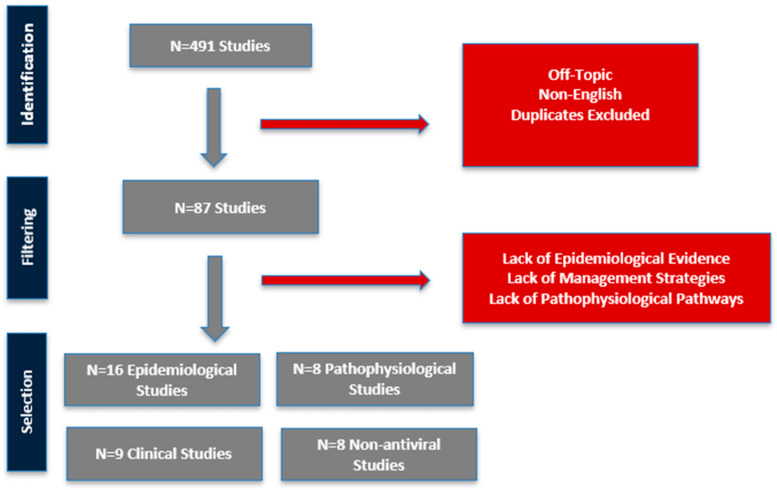
Workflow for filtering included studies.

The initial database search returned 491 results, from which 404 were excluded based on titles and abstracts for being off-topic, duplicative, or non-original. Full-text review of 87 articles led to the final inclusion of 41 studies that directly addressed one or more of the four research domains: (1) epidemiological studies, (2) pathophysiological pathways linking VZV to stroke, (3) clinical models of infection and (4) non-antiviral strategies (Figure 1).

## 3. Results

The initial database search returned 491 records, which were systematically filtered through title, abstract, and full-text review. This process culminated in the inclusion of 41 studies that directly addressed the objectives concerning the VZV–stroke relationship, its underlying mechanisms, and potential management strategies. The findings from these studies are synthesized and organized into four distinct tables for detailed analysis.

Table 1 consolidates evidence from 16 epidemiological studies conducted across North America, Europe, and Asia, providing robust, population-level data on the association between VZV infection and cerebrovascular events [36,37,38,39,40,41,42,43,44,45,46,47,48,49,50,51,52].

The most consistent finding across these studies is a time-dependent escalation in stroke risk following an episode of HZ. A seminal systematic review and meta-analysis [36], encompassing 27 individual studies, quantified this risk with precision, demonstrating a peak relative risk of 1.80 within the first 14 days post-HZ [36]. This risk remained significantly elevated over time. This acute risk phase was corroborated by large-scale national cohort studies. For instance, a Danish cohort of 4.6 million adults reported a 126% increase in stroke risk within the first two weeks, while a US study of veterans found 93% higher risk of stroke within 30 days of a zoster diagnosis [37,40].

Beyond the immediate period, several studies indicate that the risk may persist long-term. A prospective cohort of over 200,000 US health professionals reported elevated risk of cardiac pathology post-infection, peaking at 5–8 years post-infection and remaining elevated for up to 12 years [44]. The epidemiological data also reveal important high-risk subgroups. Immunocompromised populations, particularly those with HIV, face a substantially heightened risk, i.e., evidence of CNS VZV reactivation in 37% of HIV−positive stroke patients in South Africa [38]. Furthermore, contrary to the assumption that age is the primary risk factor, several studies indicate that younger individuals experience a disproportionately higher relative risk. Sundström et al. reported an incidence rate ratio in patients aged 0–39 years vastly higher than that observed in older cohorts [52]. This trend was echoed in a Korean study in patients under 40 [47]. The risk extends to pediatric populations following primary varicella infection, reporting elevated incidence rate during the first half of the year following chickenpox [43].

Recent studies provide more granular insights into whether VZV infection or reactivation confers different risks for ischemic versus hemorrhagic stroke. A landmark retrospective cohort analysis from the UK reported that ischemic stroke was more commonly reported (33%) compared to hemorrhagic stroke (6%) [50]. Similarly, other studies found that the majority of cerebrovascular events occurring after infection were ischemic in nature [4,41]. Temporal patterns also diverge as the incidence of ischemic stroke peaks within the first month after zoster and gradually declines thereafter, while hemorrhagic stroke tends to appear later [4,48].

Table 2 synthesizes findings from 8 clinicopathologic studies that move beyond association to delineate the precise mechanisms by which VZV induces cerebrovascular disease [53,54,55,56,57,58].

A cornerstone of the causal argument is the repeated direct detection of the virus within affected cerebral arteries. The foundational work by Gilden et al. provided the first clear evidence, identifying VZV DNA and antigens in the cerebral arteries without the presence of a skin rash [53]. Subsequent research has consistently replicated this finding [14]. The proposed pathogenic trajectory involves the reactivated virus traveling transaxonally from cranial nerve ganglia (particularly the trigeminal ganglion) to the adventitia (outer layer) of cerebral arteries [54,56]. From this initial site of infection, the virus instigates a transmural inflammatory cascade, spreading inward and causing a characteristic vasculopathy. The histopathological hallmarks of this process, confirmed in post-mortem analyses, include intimal hyperplasia, medial smooth muscle loss, and granulomatous inflammation, often with multinucleated giant cells.

These structural changes have direct clinical consequences, leading to arterial stenosis, thrombosis, aneurysm formation, and ultimately, vessel rupture or occlusion, manifesting as ischemic or hemorrhagic stroke. Advanced imaging techniques, particularly high-resolution vessel wall MRI, have allowed for the in vivo visualization of these inflammatory changes, providing a radiological correlate to the pathological findings [19,58].

A critical and sophisticated aspect of VZV vasculopathy is the virus’s ability to evade host immune surveillance. Experimental work reported that post-infection upregulation of human cerebral vascular adventitial fibroblasts leads to the downregulation of key immune molecules, including Programmed Death-Ligand 1 (PD-L1) and Major Histocompatibility Complex class I (MHC-I) [55]. This impairment of immune clearance mechanisms allows for viral persistence within the arterial wall, fostering a state of chronic inflammation and vascular remodeling.

Table 3 compiles data from 9 clinical studies and reviews that evaluate the effectiveness of various interventions on stroke-related outcomes, revealing a nuanced and sometimes contradictory picture [40,59,60,61,62,63,64,65,66].

The efficacy of antiviral therapy in preventing stroke post-HZ is a subject of ongoing debate. While in vitro data and clinical practice support their use for treating active infection, their role in mitigating long-term vascular complications is less clear. A meta-analysis of 12 observational studies concluded that antiviral treatment did not confer a significant protective effect against stroke [63]. This contrasts with findings from Kawai et al., who analyzed a national database of millions and found that antiviral prescription within 7 days of HZ onset was associated with an approximately 21% reduction in stroke risk, indicating that timing may be a critical factor [61]. In pediatric populations, the evidence is also mixed; Lewandowski et al. found no clear evidence that antiviral treatment altered the disease course in immunocompetent children with CNS VZV disease [66].

In contrast to the ambiguous data on antivirals, the protective effect of vaccination is far more consistent and compelling. The same meta-analysis by Jia et al. found that zoster vaccination led to a 22% lower probability of stroke risk [63]. This positions vaccination as the most effective primary prevention strategy for VZV-associated stroke, a conclusion strongly supported by additional studies [64].

For patients who have already developed VZV vasculopathy, combination therapy appears to be key. Promising evidence for the adjunctive use of corticosteroids comes from case series that documented three young men with VZV vasculitis and intracranial stenoses [62]. All three patients showed significant or near-complete resolution of arterial stenoses after a prolonged course of valacyclovir combined with a slow taper of oral prednisolone, with no recurrent strokes. However, the limitations of this approach are highlighted by other case reports, which detailed a fatal outcome despite combined antiviral and high-dose steroid therapy, underscoring the potential for poor prognosis in severe cases, particularly in immunocompromised hosts [65].

Table 4 includes 8 studies investigating compounds that operate outside the traditional antiviral mechanism, highlighting a diverse and evolving research frontier [28,67,68,69,70,71].

The most clinically established non-antiviral approach is the use of immunomodulatory agents, specifically corticosteroids (e.g., prednisone, methylprednisolone) [28,62,69,70]. These are widely used as adjuncts to antivirals in severe VZV vasculopathy to suppress the damaging inflammatory response within the vessel wall, although their use is tempered by concerns of immunosuppression.

Beyond repurposed drugs, significant research is focused on novel agents. Several natural compounds, including Myricetin, Apigenin-4′-glucoside, and Abyssinone V, have been identified through in silico modeling as potential inhibitors of VZV thymidine kinase, a key viral enzyme [67]. However, these remain in the preclinical investigation stage. Another promising avenue is host-directed therapy. The natural polyphenol Resveratrol [71], with its known vasodilatory, antioxidant, and anti-inflammatory properties, has been proposed as a potential neuroprotective agent in VZV-induced vasculopathy, though clinical validation is pending.

## 4. Discussion

A growing body of research underscores an intricate relationship between VZV infection/reactivation and stroke. Epidemiological studies consistently show that individuals who experience herpes zoster have a significantly elevated risk of cerebrovascular events, especially in the early period following the infection [38]. Available epidemiological datasets regularly demonstrate that the association between VZV and stroke is driven primarily by ischemic events, whereas hemorrhagic strokes occur far less frequently [4]. Meta-analyses report that stroke incidence is higher in the first two weeks after a zoster episode, with the excess risk gradually attenuating over subsequent months [38]. This temporal pattern strongly suggests a causal trigger: VZV reactivation acts as a stroke precipitant, particularly in the short term. Clinical observations mirror these findings. Case series have documented strokes occurring within days to weeks of shingles, including instances where patients developed vasculitic stroke in the absence of dermatological signs [72]. Such reports highlight that VZV’s impact on the vasculature can manifest covertly, complicating straightforward clinical recognition. Taken together, epidemiological and clinical evidence converge to identify VZV as a non-traditional yet important risk factor for stroke. Notably, this association spans diverse patient populations: older adults with shingles comprise the largest at-risk group, but pediatric strokes are also well documented [38].

Mechanistic studies provide a biological framework explaining how VZV induces cerebrovascular damage. VZV is neurotropic and vasculotropic: after reactivation in cranial nerve ganglia, the virus can spread transaxonally to infect cerebral arteries, particularly those innervated by branches of the trigeminal nerve [72]. Pathological analyses confirm direct viral invasion of arterial walls—VZV DNA, proteins, and even viral inclusion bodies have been found in intracranial arteries from stroke patients with prior zoster [72]. Within the vessel, VZV triggers a vasculitis characterized by transmural inflammation and profound structural remodeling. These changes lead to marked intimal thickening and luminal narrowing, predisposing to thrombosis and ischemia [72]. VZV reactivation provokes a systemic inflammatory response with elevated cytokines and can generate prothrombotic autoantibodies and immune complexes [72]. These factors may promote a hypercoagulable state and endothelial dysfunction, compounding the risk of stroke.

Human dorsal root ganglion neurons and in vivo models have shown that VZV reactivation in sensory ganglia leads to anterograde transport of virus along axons toward peripheral and cerebral arteries [73,74,75]. The viral glycoprotein complex gE–gI plays a critical role in sorting virions into neuronal processes and enabling efficient cell-to-cell spread [73,74,75]. Although axonal transport mechanisms are well characterized in other alphaherpesviruses such as HSV-1 and pseudorabies virus—where US9, gE/gI, and kinesin motors mediate anterograde trafficking—the precise molecular machinery used by VZV remains less fully defined [73,74,75]. Once VZV reaches perivascular nerve terminals, studies in human brain vascular tissue demonstrate that the virus infects adventitial fibroblasts, which subsequently activate endothelial cells, release inflammatory mediators, and promote transmural spread, providing a mechanistic basis for VZV vasculopathy [73,74,75]. Jones et al. demonstrated that VZV infection of human brain vascular adventitial fibroblasts, perineurial cells, and lung fibroblasts leads to a marked reduction in PD-L1 and MHC-I surface expression within 72 h [76]. Importantly, this suppression occurs post-transcriptionally, as PD-L1 mRNA levels remain unchanged, implicating virally induced protein destabilization or altered trafficking [76]. This reduction in MHC-I disrupts CD8^+^ T-cell recognition of infected vascular cells, enabling viral persistence in the arterial wall [76]. In contrast, the loss of PD-L1 removes a key inhibitory checkpoint that normally limits T-cell activation, resulting in heightened local cytokine release (e.g., IFN-γ, TNF-α) and sustained vascular inflammation [76]. Hence, VZV causes a paradoxical state of simultaneous immune evasion and unrestrained inflammation, which supports chronic vasculopathy.

From a management standpoint, identifying VZV involvement is crucial because it directs specific therapy. The mainstay treatment for VZV vasculopathy is antiviral therapy. High-dose intravenous acyclovir is recommended to suppress VZV replication [64]. This is often initiated empirically in suspected cases, given the disease’s severity and the time sensitivity of stroke management. Adjunctive corticosteroids are commonly co-administered with antivirals, aiming to reduce vessel wall inflammation and swelling [64]. The adjunctive use of corticosteroids in VZV vasculopathy presents a critical risk-benefit dilemma centered on the balance between suppressing harmful arterial inflammation and risking immunosuppression-related complications. The anti-inflammatory benefit, demonstrating resolution of intracranial stenoses with combined antiviral and steroid therapy, must be weighed against the significant hazard of impaired viral clearance and opportunistic infections [62]. This risk is not uniform, as it is considered acceptable in immunocompetent patients with severe, progressive large-vessel disease, where the threat of infarction is imminent, but is unacceptably high in immunocompromised hosts, where steroid use may precipitate fatal outcomes [65]. Consequently, a pragmatic approach is to reserve corticosteroid therapy for immunocompetent adults, initiating treatment with high-dose intravenous methylprednisolone alongside antivirals. Its use in immunocompromised patients or in mild or pediatric cases should be avoided or undertaken with extreme caution under close monitoring, acknowledging that current studies are based on low-level evidence. Finally, it is important to note that standard anticoagulant therapy alone does nothing to eradicate the underlying virus; thus, without antivirals, ongoing arterial injury may continue. This is illustrated by the observation that patients who do not receive antiviral treatment for zoster have higher rates of stroke than those who are treated, all else being equal [61]. Early antiviral therapy during a zoster episode led to a lower probability of stroke, supporting the practice of prompt antiviral initiation not just to hasten rash resolution but also as a stroke-preventive measure [61].

The long-term prognosis of VZV-associated stroke is variable. Studies show that stroke risk remains elevated for several months after zoster, with the highest recurrence risk occurring within the first 4–12 weeks [7,36]. Nevertheless, meaningful neurological recovery is feasible, and even patients with prolonged, multifocal VZV vasculopathy have demonstrated cognitive and gait improvement after timely antiviral therapy, including cases treated successfully after more than two years of ongoing infarcts [28]. Persistent sequelae—such as visual loss, cognitive impairment, and focal deficits—may occur but can be mitigated through antiviral treatment, risk-factor control, and standard post-stroke rehabilitation [77]. Early recognition and antiviral therapy remain the strongest determinants of long-term outcome in VZV-associated stroke.

Preventive measures, particularly vaccination, are at the forefront of reducing this VZV–stroke burden. A large Veterans Affairs study found that patients who had received zoster vaccines had significantly lower stroke rates following VZV infection [37]. In that study, prior vaccination was associated with over 40% lower risk of stroke after administering the recombinant vaccine and about a 23% reduction for the live vaccine [37]. Considering such insights, health policy should emphasize improving zoster vaccine uptake. Overall, preventive approaches—via vaccination and secondary prevention via early antiviral treatment—form a twin strategy to diminish the impact of VZV-related strokes on society.

To date, no dedicated randomized controlled trials have been conducted to determine the optimal treatment regimen for VZV-related stroke. Questions remain about the ideal duration of antiviral therapy, the role of combination therapy, and the management of steroid-responsive inflammation. Future studies should explore whether prolonged antiviral courses or suppressive antiviral prophylaxis could prevent recurrent strokes in high-risk patients. The potential benefit of anti-inflammatory or immunomodulatory adjuncts, including aspirin, statins, and calcineurin inhibitors, also needs clearer evidence and proper integration into therapeutic protocols. Exploratory approaches such as CRISPR/Cas9 gene editing and antisense technologies aimed at VZV could one day enable eradication of latent VZV or suppression of viral gene expression [32,33]. Additionally, drugs like resveratrol that exhibit both antiviral activity and neurovascular protective effects are being investigated as potential adjuncts or prophylactics against VZV-induced stroke [71].

This work has several limitations that should be acknowledged. Firstly, the inherent heterogeneity of the included studies—encompassing in vitro models, systematic reviews, and human case reports—precludes a formal meta-analysis, thus limiting the ability to quantify the overall strength of the evidence. The heavy reliance on in vitro data and the scarcity of robust, representative human studies for VZV vasculopathy create a significant translational gap and mean that the promising mechanistic findings and novel therapeutic candidates remain largely theoretical. Furthermore, clinical evidence is often derived from reports and small cohort studies, which are susceptible to publication bias and lack the controlled rigor of randomized trials; consequently, the efficacy of adjunctive therapies like corticosteroids and statins is suggested by anecdotal evidence rather than proven by high-quality data. Finally, while we underscore the paramount importance of immunization, the global inequity in vaccine access means the findings may not be generalizable to all populations.

## 5. Conclusions

Varicella Zoster Virus is a potent and direct trigger of cerebrovascular disease through a mechanism of direct viral invasion and inflammation of cerebral arteries. We consolidate the multi-faceted evidence, from epidemiology and pathology to in vitro models, that establishes this intricate relationship.

The clinical implications are profound. A high index of suspicion for VZV vasculopathy is warranted in cases of cryptogenic stroke, especially in the elderly and immunocompromised, and even in children, even in the absence of a characteristic rash. Diagnosis should prioritize CSF serology for VZV-specific antibodies, supplemented by advanced vascular imaging like high-resolution vessel wall MRI. Treatment must evolve beyond monotherapy with antivirals to consider combination strategies that address the concomitant inflammatory cascade, though more clinical trials are needed to define optimal protocols.

Ultimately, prevention remains the most effective and sustainable strategy. The recombinant zoster vaccine, with its high efficacy and favorable safety profile, offers a powerful tool to mitigate a significant portion of the burden of VZV-related stroke. Future research should focus on validating novel non-antiviral adjuncts in clinical trials and further elucidating the precise molecular mechanisms of VZV-induced vascular injury to identify new therapeutic targets. By integrating vaccination, improved diagnostics, and multi-modal treatment, the medical community can effectively confront the significant and often preventable cerebrovascular complications of VZV infection.

## Figures and Tables

**Table 1:** Epidemiological Studies Linking VZV–Stroke.

Author Year Country	Type of Study	Population	Key Findings on Stroke Risk and VZV
Lu et al. 2023 China [36]	Systematic Review and Meta-Analysis	27 studies (17 on zoster, 10 on chickenpox)	Stroke risk is significantly increased after HZ, decreasing over time: RR 1.80 (14 days), RR 1.61 (30 days), RR 1.45 (90 days), RR 1.32 (180 days), RR 1.27 (1 year).
Parameswaran et al. 2023 USA [37]	Retrospective Case–Control	2,165,505 US veterans (71,911 with zoster)	1.93× increased odds of stroke within 30 days of zoster.
Marais et al. 2022 South Africa [38]	Observational Cohort	35 HIV+ stroke patients, 34 HIV− stroke patients, 18 HIV+ controls	CNS VZV reactivation was found in 37% of HIV+ stroke patients, 9% of HIV− stroke patients, and 6% of HIV+ controls. A positive CSF VZV PCR was exclusive to HIV+ stroke patients (20%).
Salim & Hamad 2024 Iraq [39]	Observational Cross-Sectional	90 patients with stroke	VZV DNA present in about 38% patients and over 40% of controls. Higher infection rates were found in females (52.78%) and the ≥51 age group (66.67%).
Sreenivasan et al. 2013 Denmark [40]	Nationwide Cohort Study	4.6 million adults; >117,926 treated with antivirals	Stroke risk more than doubled early on and plateaued at 17% up to 12 months. Antiviral therapy was associated with a reduced risk.
Langan et al. 2014 UK [41]	Self-Controlled Case Series	6584 individuals with both zoster and stroke	Stroke risk was increased 1–26 weeks after zoster, peaking at 1–4 weeks (IRR 1.63). Antiviral therapy was associated with a reduced stroke risk.
Kang et al. 2009 Taiwan [42]	Retrospective Cohort Study	>7000 adults with zoster and >23,000 controls	Stroke risk increased by 30% in the first year after zoster. Risk was 4-fold greater with ophthalmic-distribution zoster.
Thomas et al. 2013 UK [43]	Self-Controlled Case Series	560 individuals (60 children, 500 adults) from primary care databases had both chickenpox and a stroke.	Children had a 4-fold elevated incidence rate of stroke in the half year post-infection. Adults had a less marked, but significant, increased risk (IR 2.13). Risk was no different beyond this period.
Curhan et al. 2022 USA [44]	Prospective Cohort Study	205,030 US health professionals without prior history	Risk peaked at 5–8 years post-HZ (HR 1.38) and remained elevated for up to 12 years.
Yawn et al. 2016 USA [45]	Retrospective Community Cohort Study	4862 adults aged ≥ 50 with zoster and 19,433 matched controls.	Risk of stroke at 3 months increased by 50% after adjusting for risk factors. No significantly increased risk for stroke or MI was found beyond 3 months when controlling for cardiovascular risk factors.
Seo et al., 2018, Korea [46]	Nationwide Retrospective Study	20,311 HZ patients, 13,980 CVD patients, and matched controls	Hospitalization elevated risk of stroke. Patients with prior MI, stroke, or HF were also at increased risk of subsequent severe HZ
Kim et al., 2017, Korea [47]	Population-Based Cohort with Propensity Score Matching	519,880 individuals (23,233 HZ cases; matched controls)	HZ significantly increased risk of composite CV events, including stroke. In matched analysis: HR 1.41 (composite) and HR 1.35 (stroke). Risks were highest in younger patients (<40 yrs) and during the first year post-HZ
Hosamirudsari et al., 2018, Iran [48]	Case–Control Study	105 patients	Elevated risk (OR 5.84; 95% CI 1.98–8.23). Risk was highest 2–4 weeks post-HZ. Ischemic strokes were more frequent than hemorrhagic strokes.
Schink et al., 2016, Germany [49]	Case Series (GePaRD database)	124,462 stroke patients	Stroke risk increased by 30% within 3 months post-HZ. Hemorrhagic stroke risk is higher by 53% vs. ischemic 27%. Risk declined by 6 months.
Minassian et al., 2015, USA [50]	Case Series	351,865 ≥65 yrs; 42,954 with stroke	Marked increase in first week post-HZ: IR 2.37 (stroke), IR 1.68 (MI). Risk declined over 6 months.
Kwon et al., 2016, Korea [51]	Prospective Nationwide Dynamic Cohort	766,179 adults followed for 11 years; 70,424 HZ cases, 75,932 stroke cases	Risk significantly increased risk of stroke by 90%. Adjusted HRs by age: 2.04 (18–30 yrs), 1.74 (30–40 yrs), 1.43 (40–50 yrs), 1.23 (50–60 yrs), 1.24 (60–70 yrs), 1.29 (>70 yrs). Elevated risk persisted for several years.
Sundström et al., 2015, Sweden [52]	Population-Based Cohort Study	13,296 HZ cases	Stroke risk significantly increased within 1 year after HZ (IRR 1.34; 95% CI 1.12–1.62). Effect strongest in younger patients: IRR 10.3 (0–39 yrs), IRR 9.42 (40–49 yrs). In older patients, risks closer to unity (~1.3).

CI: Confidence Interval; CNS: Central Nervous System; CSF: Cerebrospinal Fluid; CVD: Cardiovascular Disease; GePaRD: German Pharmacoepidemiological Research Database; HZ: Herpes Zoster; PCR: Polymerase Chain Reaction; OR: Odds Ratio; RR: Relative Risk; IRR: Incidence Rate Ratio.

**Table 2:** Pathophysiological Studies Linking VZV–Stroke.

Author Year Country	Study Type Population/Model	Key Findings on VZV–Stroke Pathophysiology
Hoshino et al., 2019, Japan [14]	Case series Immunocompromised adults with confirmed VZV vasculopathy	All developed ischemic stroke after zoster. CSF VZV DNA/IgG positive. Cranial neuropathies are often prodromal. Thrombotic markers elevated.
Tsivgoulis et al., 2016, Greece [19]	Case report with advanced imaging 21-year-old woman with acute ischemic stroke	MRI showed ischemic lesions with ICA stenosis. CSF is positive for VZV DNA/IgG. High-resolution vessel wall MRI revealed arteritis.
Gilden et al., 1996, USA [53]	Clinicopathologic Case Report 73-year-old man with waxing/waning CNS vasculitis, no rash	Post-mortem: VZV DNA/antigen in cerebral arteries. Pathology: patchy vasculitis, IEL disruption, giant cells, infarcts. Proved VZV vasculitis can occur without rash.
Nagel, 2014, USA [54]	Clinical cases, CSF, pathology	Pathogenesis: adventitial infection → transmural spread → intimal thickening, IEL disruption, smooth muscle loss. CSF anti-VZV IgG more sensitive than PCR.
Jones et al., 2016, USA [55]	Experimental virology Human vascular adventitial fibroblasts and perineurial cells	VZV downregulates PD-L1 and MHC-I, impairing immune clearance. Leads to persistent inflammation and vascular remodeling → chronic vasculopathy.
Bakradze et al., 2019, USA [56]	Case series, imaging, pathology	VZV spreads from ganglia to arteries, infects adventitia, then transmurally. Causes vascular remodeling, medial necrosis, granulomatous arteritis. Intrathecal IgG synthesis is more reliable than PCR.
Yawn et al., 2022, USA [57]	Summary of 15+ years of studies	VZV reactivation spreads transaxonally to cerebral arteries. Causes vascular inflammation, remodeling, sympathetic activation. Post-mortem confirms VZV DNA/antigen in arteries. Antivirals may lower risk (evidence mixed).
Wu et al., 2022, China [58]	Case Report 67-year-old man, VZV meningoencephalitis → stroke	CSF PCR positive for VZV. Months later: multiple infarcts, HR-MRI showed circumferential plaques (vasculitis pattern, not atherosclerosis). Supports trigeminal → MCA adventitial spread.

CSF—Cerebrospinal Fluid; DNA: Deoxyribonucleic Acid; HR-MRI—High-Resolution Magnetic Resonance Imaging; ICA—Internal carotid artery; IEL—Internal Elastic Lamina; IgG: Immunoglobulin G; PCR—Polymerase Chain Reaction; MCA—Middle Cerebral Artery; MHC-I—Major Histocompatibility Complex class I; PD-L1—Programmed Death-Ligand 1; VZV: Varicella Zoster Virus.

**Table 3:** Clinical Studies Linking VZV-Related Stroke and Antiviral Treatments.

Study (Author, Year, Country)	Model	Treatment Agent	Outcome
Sreenivasan et al., 2013, Denmark [40]	Nationwide population-based cohort (4.6 M adults)	Antivirals (acyclovir, valacyclovir, famciclovir)	No protective effect from antivirals on long-term stroke risk.
Düzgöl et al., 2016, Tukey [59]	Retrospective cohort (41 pediatric malignancy patients with VZV)	IV acyclovir (5–21 days)	93% recovered without complications. 3 (7%) developed ARDS, 1 (2%) died (HLH). Acyclovir effective overall but mortality persisted in immunocompromised children; delayed chemotherapy a major impact.
Yang et al., 2020, USA [60]	Self-controlled case series (87,405, ≥66 yrs)	Zoster vaccine live (ZVL); antivirals (acyclovir, valacyclovir, famciclovir)	Risk of AIS increased after HZ (IRR 1.89 at 0–14 days). Neither ZVL nor antivirals significantly modified this risk. Suggests vaccination to prevent HZ is more effective than post-HZ antivirals.
Kawai et al., 2021, Japan [61]	Nationwide cohort (Japan National Database, millions of patients)	Antivirals (acyclovir, valacyclovir, famciclovir)	Antivirals prescribed within 7 days of HZ reduced risk of stroke (adjusted HR ~0.79). Stronger benefit in younger, otherwise healthy patients.
Kraemer et al., 2022, Germany [62]	Case series (3 young men, VZV vasculitis with intracranial stenoses)	Long-term valacyclovir + oral prednisolone (slow taper)	Significant resolution of MCA/ICA stenoses in all 3 patients, 2 near-complete. No recurrent strokes. Side effects from steroids only. Supports prolonged antiviral + steroid regimen for large-vessel VZV vasculitis.
Jia et al., 2023, China [63]	Systematic Review and Meta-Analysis (12 observational studies)	HZ vaccines (ZVL, RZV); antivirals	Vaccines: reduced stroke risk (OR 0.78, 95% CI 0.68–0.90). SCCS: lower risk in vaccinated by 20%. Antivirals: no protective effect (OR 1.13, 95% CI 0.94–1.36).
Yawn & Gilden, 2024, USA [64]	Review	Antivirals; Vaccines (ZVL, RZV)	Antivirals: inconsistent benefit, timing critical. Vaccines: stronger evidence—ZVL lowered stroke risk (HR ~0.84, Medicare), RZV highly protective against HZ and emerging data suggest reduced stroke. Vaccination = best prevention strategy.
Ando et al., 2025, Japan [65]	Autopsy case report (60-year-old man with VZV → vasculopathy)	IV acyclovir (renal-adjusted), oral valacyclovir, prednisolone, IV methylprednisolone	Despite antivirals + steroids, progressive ACA/MCA stenoses → multiple infarcts and death. Autopsy: IEL disruption, inflammatory infiltration, thrombosis. Highlights poor prognosis with delayed/low-dose therapy in immunocompromised patient.
Lewandowski et al., 2024, Poland [66]	Case Series (55 immunocompetent children)	Antivirals (Acyclovir, Valacyclovir, Famciclovir)	75% (41/55) of children received antiviral treatment. No clear evidence that antiviral treatment altered the disease course, as most patients (including untreated ones) recovered without complications

ACA—Anterior Cerebral Artery; AIS—Acute Ischemic Stroke; ARDS—Acute Respiratory Distress Syndrome; CSF—Cerebrospinal Fluid; CNS—Central Nervous System; HLH—Hemophagocytic Lymphohistiocytosis; HR—Hazard Ratio; ICA—Internal Carotid Artery; RZV—Recombinant Zoster Vaccine; SCCS—Self-Controlled Case Series; TIA—Transient Ischemic Attack; ZVL—Zoster Vaccine Live.

**Table 4:** Studies on VZV-related Stroke and Non-Antiviral Compounds.

Study (Author, Year, Country)	Compound	Current status	Mode of action	Key Preclinical Findings and Translational Potential
Kwofie et al., 2022, Ghana [67]	Myricetin	Under preclinical investigation	In silico predicted inhibitor of VZV thymidine kinase	Strong predicted antiviral. Molecular docking showed a binding affinity of –9.3 kcal/mol to VZV No AMES test toxicity, no hepatotoxicity Strong clinical translation potential
Kwofie et al., 2022, Ghana [67]	Apigenin-4′-glucoside	Under preclinical investigation	In silico predicted inhibitor of VZV thymidine kinase.	Docking affinity of—10.2 kcal/mol to VZV Negative AMES test/hepatotoxicity Strong clinical translation potential
Kwofie et al., 2022, Ghana [67]	Abyssinone V	Preclinical candidate	In silico binding to VZV thymidine kinase	Documented biochemical activity against other viruses—specifically pneumococcal neuraminidase Docking affinity of −9.6 kcal/mol to VZV No AMES test toxicity, no hepatotoxicity High potential translation potential
Niemeyer et al., 2024, USA [68]	Host-derived Small Extracellular Vesicles	Mechanistic discovery, no specific compound identified	VZV-infected neurons release sEVs that suppress interferon signaling, potentially worsening immune response	Strongly inhibit innate antiviral signaling (suppressing IFN-β release) without causing cytotoxicity themselves
Nagel & Gilden, 2016, USA [69]	Prednisone	On market	Adjunctive therapy in VZV vasculopathy and VZV-related giant cell arteritis	Prednisone can be clinically useful in VZV-related vasculopathy when combined with antiviral therapy, helping control inflammation while antivirals treat the underlying viral infection
Silver et al., 2012, USA [28]	Methylprednisolone	On market	High-dose IV corticosteroid for acute VZV-related vasculopathy or stroke	High-dose methylprednisolone may assist in reducing vascular inflammation in VZV vasculitis when paired with timely antiviral therapy to address the viral trigger.
Kennedy, 2016, UK [70]	Dexamethasone	On market	Considered in severe VZV CNS complications and used empirically in vasculitis	Dexamethasone can provide symptom relief and reduce inflammatory vessel damage in VZV-associated arteritis when used adjunctively with appropriate antiviral treatment.
Zhang & Wu, 2011, China [71]	Resveratrol	On market (off-label)	May prevent ischemic stroke through NO-mediated vasodilation, antioxidant effects, and inhibition of COX	Resveratrol shows strong inhibitory activity, reducing superoxide production at 30–100 mg/kg and suppressing inflammatory mediators (COX-2) in a dose-dependent manner, while demonstrating no toxicity in humans

sEVs—small Extracellular Vesicles; IV—Intravenous; NO—Nitric Oxide; COX—Cyclooxygenase; VZV: Varicella Zoster Virus.

## Data Availability

No new data were created or analyzed in this study. Data sharing is not applicable to this article.
